# Case Report: Cryptococcal meningitis in an apparently immunocompetent patient in Nepal - challenges in diagnosis and treatment

**DOI:** 10.12688/wellcomeopenres.15187.2

**Published:** 2019-06-17

**Authors:** Ashish Jha, Sudeep Adhikari, Keshav Raj Sigdel, Buddhi Paudyal, Buddha Basnyat, Gyan Kayastha, Sumita Pradhan, Ujjwol Risal, Jiwan Poudel

**Affiliations:** 1 Internal Medicine, Patan Academy of Health Sciences, Lalitpur, Nepal; 2 Oxford University Clinical Research Unit, Patan Hospital, Lalitpur, Nepal; 3 General Surgery, Patan Academy of Health Sciences, Lalitpur, Nepal

**Keywords:** cryptococcal meningitis, immunocompetent patient, liposomal amphotericin B

## Abstract

A 50 year old woman from Nepal had clinical features suggestive of meningitis. Cerebrospinal fluid (CSF) analysis was normal except for the presence of cryptococcal antigen. The inclusion of test for *Cryptococcus* in the CSF helped in making the diagnosis of cryptococcal meningitis in our patient who was apparently immunocompetent. Treatment with liposomal amphotericin B could not be started on time due to financial constraints. The patient had a stroke and further deteriorated. Liposomal amphotericin B is stocked by the government of Nepal for free supply to patients with visceral leishmaniasis, but the policy does not allow the drug to be dispensed for other infections. The family members of our patient acquired the drug within a few days from a government center using their political connections and following administering the treatment the patient improved. This case demonstrates the utility of considering cryptococcal meningitis as a differential diagnosis, and including tests for *Cryptococcus* when dealing with immunocompetent patients presenting with meningitis. It also demonstrates the effects of the sociopolitical situation on health care delivery in low- and middle-income countries (LMICs) such as Nepal.

## Introduction

*Cryptococcus* are encapsulated yeast, an opportunistic fungal pathogen that may lead to life- threatening infections such as meningoencephalitis and disseminated cryptococcosis, usually in immunocompromised hosts ^1^. Immunocompromised patients presenting with features of meningitis will usually be tested for *Cryptococcus* as a causative agent ^2^, but it is seldom considered when dealing with patients who are immunocompetent. Here we present a case of a patient who was apparently immunocompetent, yet developed cryptococcal meningitis. The inclusion of cryptococcal antigen testing in the initial cerebrospinal fluid (CSF) test panel led to the early diagnosis. But the management became complicated when socioeconomic and political aspects came into play. This case demonstrates how sociopolitical conditions may interfere with treatment in low- and middle-income countries (LMICs) and affect the health system of the country.

## Case presentation

A 50 years old Aryan female from Lalitpur district, Nepal, presented to Patan Hospital Emergency Department in December 2018 with complaints of abdominal pain that had persisted for two days. She had multiple episodes of vomiting. In terms of the patient’s history, she had been treated with amlodipine and atenolol for hypertension and undergone a total abdominal hysterectomy 5 years prior for fibroids. She was a farmer living in suburban area of Lalitpur and involved in growing of vegetables and rearing of cows and poultry. On assessment, her blood pressure was 170/100 mmHg and her pulse rate was 88 beats per minute. Temperature was 98 degree Fahrenheit. Abdominal examination showed tenderness in the right upper quadrant.

Below are the laboratory parameters with normal range in parentheses:

Complete blood count : white cell count 8.4 (4-10) X 10^9/L ; neutrophils 60 % ; lymphocytes 37 % ; monocyte 1 % ; eosinophil 2 % ; red blood cells 4.8 (4.2–5.4) × 10^12/L; hemoglobin 12.5 ( 12-15) g/dL ; platelets 145 ( 150-400) X 10^9/L

Biochemistry: random blood sugar 124 (79-160) mg/dL; urea 28 (17-45) mg/dL; creatinine 0.7 (0.8-1.3) mg/dL; sodium 135 (135-145) mmol/L; potassium 3.8 (3.5 – 5) mmol/L; calcium 8.5 (8.4-10.2) mg/dL; albumin 3.4 (3.5-4) gm/dL; magnesium 1.8 (1.6-2.3 mg/dL); amylase 45 (30-125) U/L; lipase 56 (10-150) U/L.

Hepatic panel: bilirubin total 2 (0.1-1.2) mg/dL and direct 0.5 (0-0.4) mg/dL; alanine transaminase 53 (5-30) units/L; aspartate transaminase 47 (5-30) units/L; alkaline phosphatase 76 (50-100) IU/L

Ultrasound of the abdomen revealed a gallstone with a thickened gall bladder wall. She was admitted to the surgical ward with a provisional diagnosis of biliary colic, and managed conservatively with intravenous fluids, anti-emetics and analgesics. Her pain subsided. and her blood pressure also fell down to normal range with analgesia. But she developed a headache from 2 ^nd^ day and fever of 101 degrees Fahrenheit on the 4 ^th^ day. Injection ceftriaxone 1 gram twice daily was started empirically. The next day, she developed altered sensorium. Neurological examination revealed disorientation to time, place and person with neck stiffness but no papilledema or any focal neurological deficit. Plantar reflex was normal bilaterally.

Plain computed tomography (CT) of the head was normal. Cerebrospinal fluid (CSF) analysis revealed the following (with normal range in parentheses): Red blood cells= 6 (0–10)/mm^3; white blood cells = absent (0–5/mm^3); protein= 45 (20–45) mg/dL; sugar= 71 (45–80) mg/dL; lactate= 2 (1.1–2.8) mmol/L; adenosine deaminase (ADA) = 6.2 U/L; GeneXpert was negative for Mycobacterium Tuberculosis; CSF India ink staining was negative; Bacterial culture showed no growth over 48 hours. However, CSF cryptococcal antigen was positive by latex agglutination (Latex-Cryptococcus Antigen Detection System; IMMY). Serological test for HIV were negative.

With the diagnosis of cryptococcal meningitis, she was transferred to the intensive care unit (ICU) and liposomal amphotericin B (4 mg/kg/day) and Fluconazole (800 mg daily) were prescribed while ceftriaxone was continued. Unfortunately her family members could not afford the expensive liposomal amphotericin B. The government of Nepal provides liposomal amphotericin B (single dose of 10 mg/kg) free of cost to patients with visceral leishmaniasis (VL) ^3^. We wrote an application letter to the relevant authority to request liposomal amphotericin B for the patient. We asked for a total of 56 vials of the medicine (each vial contained 50 mg of liposomal amphotericin B) that would be sufficient for a two-week course. The relevant government authority refused to provide liposomal amphotericin B to our patient stating that the drug is to be dispensed to patients with VL only, as per the government policy. She was then started on fluconazole alone, but her status deteriorated. In addition, she also developed right sided hemiparesis 3 days following the diagnosis. Magnetic resonance imaging (MRI) of the brain was performed that revealed an acute infarct involving the left parietal lobe and basal ganglia, with multiple acute lacunar infarcts in the bilateral frontal and parietal lobes and bilateral cerebellum. She was in sinus rhythm. Echocardiography was normal. Aspirin 75 mg and atorvastatin 40 mg daily were administered as a result.

In a few days, her family members provided the liposomal amphotericin B. They revealed that they had connections with influential politicians who helped them acquire the medicine from the same government center who had previously refused them. She then received the two-week course of liposomal amphotericin B along with fluconazole. The patient improved gradually. She had hypokalemia during treatment with Amphotericin B which was managed with a potassium chloride infusion at 5 mEq/hour. She started showing improvement in her higher mental functions after the 5th day of amphotericin B administration.

After completion of 2 weeks of Amphotericin B, her higher mental function had returned to normal, and she could walk on her own with some residual weakness in her right upper limbs. She was then discharged from hospital and daily fluconazole 800 mg was continued for 8 more weeks.

On first follow up 1 week following discharge she was doing well. She was last seen in February 2019 after completing 8 weeks of fluconazole 800 mg daily. She was asymptomatic and was prescribed a daily tablet of fluconazole 400 mg for 1 year.

## Discussion

Here we presented a case of a female patient with symptoms and signs suggestive of meningitis, who was diagnosed to have cryptococcal meningitis based on finding of cryptococcal antigen in her CSF. She had no known immunodeficiency condition such as HIV, diabetes, chronic renal or liver disease, steroid or other immunosuppressive drug use. Though subclinical immunodeficiency was not ruled out, we could say that she was apparently immunocompetent. Based on the fact that cryptococcal meningitis is not uncommonly seen even in immunocompetent patients ^4^, in our hospital we routinely send out cryptococcal antigen testing in the meningitis test panel. Otherwise in most hospitals in Nepal (indeed in South Asia) the usual management protocol employed for immunocompetent persons with meningoencephalitis includes considering testing and treating for fungal infections like cryptococcus only when they fail to respond to usual antibiotics and antiviral treatment.

Cryptococcus grows readily in soil contaminated with avian excreta particularly that of pigeons, and is transmitted to human via inhalation of the contaminated aerosol. One of the possibilities of transmission in our patient may be this as she is a farmer and the Lalitpur area is home for many pigeons too. However, it is equally possible that the fungus might have been transmitted via other sources such as vegetables, fruits and dairy products.

The CSF findings in our patient was normal except for the positive cryptococcal antigen test. Cryptococcal meningitis patients with HIV infection may have normal CSF cell count, protein and sugar as there may not be adequate inflammation in the CSF to be detected. Though extremely uncommon, there are examples where patients without HIV infection have low CSF cell counts. In 1996, Khanna *et al.* reported 3 out of 23 HIV negative patients with cryptococcal meningitis had low CSF cell count ^5^. The India ink staining for fungus was negative in our patient, this test is only 30–50% sensitive in HIV negative patients, while in HIV-positive patients its sensitivity increases to 80% due to higher fungal loads in the CSF ^6^. CSF culture for *Cryptococcus* is considered the gold standard for diagnosis but has several disadvantages. It takes about 7 days for fungus to grow resulting in a delay in therapy starting ^7^, additionally, this test is not readily available in Nepal and therefore was not performed. Even if performed, there was a high possibility of it being negative because of the low fungal load in the CSF of our patient, as evidenced by the negative India ink stain. The sensitivity and specificity of the antigen testing by latex agglutination range from 93–100% and 93–98% respectively ^8^, hence we relied on its positive result for making the diagnosis. Had we not performed this test on our patient, the diagnosis would have been easily missed due to the normal CSF parameters with a negative India ink stain. It is important to keep this diagnosis in mind in a patient with altered sensorium and neck stiffness.

Cryptococcal meningitis is a life-threatening disease and fatal without treatment. Liposomal amphotericin B and Flucytosine combination treatment is the standard of care ^9^, however, these medicines are expensive making it difficult for patients from countries such as Nepal to afford the treatment on their own, as in our patient. We substituted Fluconazole for Flucytosine as it is cheaper. But without the addition of amphotericin B, Fluconazole would not have cured her. When we started fluconazole alone, she developed right hemiparesis and her status deteriorated. Cryptococcal meningitis can cause multiple infarcts in brain as a complication leading to focal neurological deficits ^10^. She improved after adding liposomal amphotericin B.

The clinical picture in our patient was little atypical for cryptococcal meningitis (apparently immunocompetent person with acute neurological deterioration and normal CSF apart from latex agglutination). So the possibility of the antigen test being a false positive result was strongly considered. However, we made the diagnosis of cryptococcal meningitis based on detection of cryptococcal antigen in CSF and lack of evidence for alternate diagnoses. Without liposomal amphotericin B, her neurological status deteriorated and developed multiple infarcts, which improved only after adding the medicine. This further strengthened our diagnosis. The difficulty in explaining the results of serological tests in the absence of standard tests such as culture can cause a delay in diagnosis and treatment in atypical clinical scenario like in our case, hence fungal culture should be made easily available for overcoming this deficit.

The government of Nepal provides liposomal amphotericin B free of cost to patients with visceral leishmaniasis (VL) ^3^, and the government policy dictates use of the medicine in patients with VL only. However the medicine is also a potent antifungal agent which is a valuable component of treatment of most of the invasive fungal infections like cryptococcosis, mucormycosis, aspergillosis which are life threatening illnesses ^11^. Nepal is an endemic region for VL with incidence of 0.11 cases per 10000 population ^12^. Each 50 mg vial of liposomal amphotericin B costs ranging 35–50 dollars, but WHO has arranged each vial at subsidized rate of 20 dollars for the use for VL ^13^. The cost of treatment of a 50 kg patient would be 200 dollars for VL which has been provided free of cost with the help from WHO. Cryptococcal meningitis is not an uncommon infection in Nepal as compared to VL with estimated burden of 0.06 cases per 10000 population ^14^. But the expenditure for its treatment is almost 6 times more (1120 dollars for a 50 kg patient) as compared to VL even if we calculate using the WHO subsidized rate. So this has been a major hindrance in treatment of this fungal infection. We did not use any standard socioeconomic scale to determine if our patient was poor. However, based on the fact that the expenditure for complete treatment of cryptococcal meningitis exceeds the annual income of an average Nepalese person (Per Capita Income of Nepal is 800 dollars as of 2017) ^15^, we assume that the inability of the family members to buy liposomal amphotericin B on their own is valid. Moreover the expensive investigations and stay in the Intensive Care Unit further hampered their ability to buy the required medicine as there is no point- of- care health insurance schemes for most patients including in our patient’s case.

In the past we have felt helpless when poor patients with such fungal infections died without the liposomal amphotericin B. If the policy does limit the use of the free supply of this medicine to patients with visceral leishmaniasis only, the government needs to come up with health insurance schemes to support the treatment of such needy patients.

It was a great relief for us and our patient when the patient’s family were able to acquire the necessary medicine. The scenario, however, was frustrating in the sense that the patient needed political connections to save her life. Our patient could potentially have died without political connection and access to liposomal amphotericin B. Corruption in health care has been a major concern in South Asia including Nepal ^16– 18^. The use of influential connections to get benefits has become ingrained in people’s attitudes ^19^. The concerned authority should establish a transparent policy so that all patients may be able to obtain the required treatment.

## Conclusion

Cryptococcal meningitis should be a diagnostic consideration even when dealing with meningitis in immunocompetent patients. Cryptococcal antigen test can help us clinch the diagnosis in time though culture would be a better diagnostic tool if available. However in Nepal, the management of this disease is complicated because liposomal amphotericin B which is the backbone of treatment, is not easily available to needy patients. The drug is in stock with the government for free supply to patients with visceral leishmaniasis. The government policy that dictates the drug to be dispensed to patients with visceral leishmaniasis only needs to be revised in order to save the lives of patients with invasive fungal infections such as cryptococcal meningitis.

## Consent

Written informed consent for publication of clinical details was obtained from the patient herself.

## Data availability

All data underlying the results are available as part of the article and no additional source data are required.
