# Supplementary material for: Case Report: Cryptococcal meningitis in an apparently immunocompetent patient in Nepal - challenges in diagnosis and treatment
Source: Wellcome Open Res. 2019 Jun 17;4:55. Originally published 2019 Mar 26. [Version 2] doi: 10.12688/wellcomeopenres.15187.2 (PMC6600853; doi:10.12688/wellcomeopenres.15187.2)
Supplement: Peer review history

# Peer review: Reviewer response for version 2

**DOI:** 10.21956/wellcomeopenres.16736.r35780

**Published:** 2019-06-26

**Authors:** Fiona Cresswell

**Affiliations:** 1 London School of Hygiene and Tropical Medicine, London, UK

In this revised version the authors have acknowledged that false positives can occur with the latex agglutination assay and the reasons why they were inclined to believe this was a true positive in this clinical scenario. 

 Access to life-saving anti-fungals is a major issue in many settings and one that needs a coordinated approach and ministry of health prioritisation to overcome. Thankfully, liposomal amphoteracin will become a little more affordable in the near future in countries eligible for concessional pricing, but there is still a long way to go. 

 Fungal culture is not feasible in many public hospital settings but the IMMY CrAg lateral flow assay (LFA) has higher specificity than the latex agglutination and can yield bedside results. Its use should be prioritised in the authors setting.

I confirm that I have read this submission and believe that I have an appropriate level of expertise to confirm that it is of an acceptable scientific standard.

# Peer review: Reviewer response for version 2

**DOI:** 10.21956/wellcomeopenres.16736.r35779

**Published:** 2019-06-20

**Authors:** Paras K. Pokharel, Lila B. Basnet

**Affiliations:** 1 School of Public Health and Community Medicine, B.P. Koirala Institute of Health Sciences, Dharan, Nepal

The revised version of the case report has sufficiently addressed our concerns and it would be appropriate to index this report to make it accessible to wider audiences.

We confirm that we have read this submission and believe that we have an appropriate level of expertise to confirm that it is of an acceptable scientific standard.

# Peer review: Reviewer response for version 1

**DOI:** 10.21956/wellcomeopenres.16569.r35654

**Published:** 2019-06-05

**Authors:** Fiona Cresswell

**Affiliations:** 1 London School of Hygiene and Tropical Medicine, London, UK

Dr Jha and colleagues present a well written case of an HIV-negative lady with suspected cryptococcal meningitis and highlight important challenges in accessing amphotericin in Nepal. The case demonstrates how socioeconomic and socio-political factors play an important role in health outcomes.

 This case presents another important real-world challenge of how to interpret imperfect tests in the absence of a gold standard. Cryptococcal meningitis is rare but recognised in HIV-negative adults and I applaud the clinicians for thinking of and testing for atypical causes of meningitis. My concern is that Cryptococcal latex agglutination is prone to false positives due to contamination or cross-reactivity, so a positive result must be interpreted with caution in a low risk population. Latex agglutination is generally recommended to be used as a screening test that is confirmed with fungal culture. Whilst I recognise that fungal culture was not available in this Nepali Hospital, it would add credibility if a positive cryptococcal antigen latex agglutination result was also confirmed on blood or indeed repeated on CSF? What type/manufacturer of kit was used? Cryptococcal antigen latex agglutination kits have a sensitivity of 93-98%, thus in a low risk population the probability of a 'positive' result being a 'false positive' may equal the probability of it being a 'true positive'. Hence the result must be applied with caution and considered in the context of the clinical picture, which in this case was a little atypical for cryptococcal meningitis.

 This 50-year-old hypertensive lady presented with biliary colic (and features of hyperbilirubinaemia and transaminitis on liver function tests) which was followed by acute confusion, fever and meningism. Cryptococcal meningitis classically has a sub-acute presentation with headache over days or weeks before onset of altered mental status. The largest review of cryptococcosis in ‘immunocompetent’ hosts has a median time to diagnosis of 68 days. Additionally, 'immunocompetent' hosts with cryptococcal meningitis usually have a white cell pleocytosis on CSF analysis, though I acknowledge acellular presentations can rarely occur. This lady’s neurological deterioration was unusually acute and CSF analysis revealed an acellular CSF with normal protein, glucose, lactate and negative India Ink.

 Three days later she developed a right hemiplegia and MRI revealed multiple acute infarcts (having had a normal CT head previously). Had she gone into atrial fibrillation in response to her acute biliary issues resulting in the multiple infarcts? What was the trajectory of her blood pressure during admission? I wonder whether this lady’s presentation have been explained by cholangitis, +/- hypertensive encephalopathy, +/- delerium and cerebrovascular accident? Did she receive broad spectrum antibiotics that might have treated biliary sepsis and delirium?

 Overall, I think this is a valuable case to present but I think the report would be more balanced if the authors acknowledged the possibility of this being a false positive result, and what steps can be taken in their setting to confirm or refute the latex agglutination result.

I confirm that I have read this submission and believe that I have an appropriate level of expertise to confirm that it is of an acceptable scientific standard, however I have significant reservations, as outlined above.

# Response

**Published:** 2019-06-11

**Authors:** Sudeep Adhikari

**Affiliations:** Patan Academy of Health Sciences, Nepal

Thank you for the constructive feedback to our article.

Below are the point by point responses to your comments. Relevant changes have been made in the case presentation and discussion parts of the first version of this article based on these responses.

**Comment**

This case presents another important real-world challenge of how to interpret imperfect tests in the absence of a gold standard. Cryptococcal meningitis is rare but recognised in HIV-negative adults and I applaud the clinicians for thinking of and testing for atypical causes of meningitis. My concern is that Cryptococcal latex agglutination is prone to false positives due to contamination or cross-reactivity, so a positive result must be interpreted with caution in a low risk population. Latex agglutination is generally recommended to be used as a screening test that is confirmed with fungal culture. Whilst I recognise that fungal culture was not available in this Nepali Hospital, it would add credibility if a positive cryptococcal antigen latex agglutination result was also confirmed on blood or indeed repeated on CSF? What type/manufacturer of kit was used? Cryptococcal antigen latex agglutination kits have a sensitivity of 93-98%, thus in a low risk population the probability of a 'positive' result being a 'false positive' may equal the probability of it being a 'true positive'. Hence the result must be applied with caution and considered in the context of the clinical picture, which in this case was a little atypical for cryptococcal meningitis.

**Answer-**

The Cryptococcal antigen latex agglutination test was done in our patient’s **CSF** by Latex-Cryptococcus Antigen Detection System; IMMY.

**Comment**

This 50-year-old hypertensive lady presented with biliary colic (and features of hyperbilirubinaemia and transaminitis on liver function tests) which was followed by acute confusion, fever and meningism. Cryptococcal meningitis classically has a sub-acute presentation with headache over days or weeks before onset of altered mental status. The largest review of cryptococcosis in ‘immunocompetent’ hosts has a median time to diagnosis of 68 days. Additionally, 'immunocompetent' hosts with cryptococcal meningitis usually have a white cell pleocytosis on CSF analysis, though I acknowledge acellular presentations can rarely occur. This lady’s neurological deterioration was unusually acute and CSF analysis revealed an acellular CSF with normal protein, glucose, lactate and negative India Ink.

**Answer-**

The clinical picture in our patient was little atypical for cryptococcal meningitis (apparently immunocompetent person with acute neurological deterioration and normal CSF apart from latex agglutination). So the possibility of the antigen test being a false positive result was strongly considered. However, we made the diagnosis of cryptococcal meningitis based on detection of cryptococcal antigen in CSF and lack of evidence for alternate diagnoses.

**Comment**

Three days later she developed a right hemiplegia and MRI revealed multiple acute infarcts (having had a normal CT head previously). Had she gone into atrial fibrillation in response to her acute biliary issues resulting in the multiple infarcts? What was the trajectory of her blood pressure during admission? I wonder whether this lady’s presentation have been explained by cholangitis, +/- hypertensive encephalopathy, +/- delerium and cerebrovascular accident? Did she receive broad spectrum antibiotics that might have treated biliary sepsis and delirium?

**Answer-**

Without the liposomal amphotericin B, her neurological status deteriorated and developed multiple infarcts. She was in sinus rhythm and her echocardiography was also normal. So the development of ischemic stroke could not be attributed to cardio-embolic phenomenon (such as atrial fibrillation). Her blood pressure was in normal range after pain relief, even without the use of antihypertensive drug (hence the possibility of hypertensive encephalopathy causing acute neurological deterioration was ruled out). She had received intravenous ceftriaxone after she developed fever, but her neurological status deteriorated which improved only after adding liposomal amphotericin B. This further strengthened our diagnosis of cryptococcal meningitis.

**Comment**

Overall, I think this is a valuable case to present but I think the report would be more balanced if the authors acknowledged the possibility of this being a false positive result, and what steps can be taken in their setting to confirm or refute the latex agglutination result.

**Answer-**

The difficulty in explaining the results of serological tests in the absence of standard tests such as culture can cause delay in diagnosis and treatment in atypical clinical scenario like in our case, hence fungal culture should be made easily available for overcoming this deficit.

# Peer review: Reviewer response for version 1

**DOI:** 10.21956/wellcomeopenres.16569.r35446

**Published:** 2019-05-20

**Authors:** Paras K. Pokharel, Lila B. Basnet

**Affiliations:** 1 School of Public Health and Community Medicine, B.P. Koirala Institute of Health Sciences, Dharan, Nepal

Some comments on Case Report:

1. The title says: “Case Report: Cryptococcal meningitis in an immunocompetent patient in Nepal - challenges in diagnosis and treatment.” However, the discussion part mentions “Though subclinical immunodeficiency was not ruled out, we could say that she was apparently immunocompetent.”
  In addition, the patient attended the hospital through emergency department and was diagnosed and treated initially for biliary colic and later developed signs of meningitis.
  Thus, it will be appropriate to write ‘apparently immunocompetent’ in the title itself.
2. Ethical Dilemma
  The authors say that they felt helpless in the past when poor people with such fungal infections died without amphotericin B.(Discussion section-4 ^th^ paragraph). This raises the ethical dilemma as public funded hospital through government system has the provision to treat free of cost if treatable disease in Nepal. There is no explanation why there were no case report when the poor patients died due to non-availability of liposomal amphotericin. As this case report displays one successful management after many deaths, can it be said that if the patient did not have any political connection she would have died without liposomal amphotericin B and this case report would not have appeared?
3. The article is focused on two issues:
  a) Cryptococcal meningitis should be a diagnostic consideration even when dealing with meningitis in immunocompetent patients.
  b) How sociopolitical conditions interfere with treatment in low- and middle-income countries (LMICs) and affect the health system of the country.
  Though these two are presented as pertinent issues that affect the disease outcome.
4. The case report has missed few important points:
  The case is described in the article as: “A 50 years old Aryan female farmer, from Lalitpur district, Nepal, presented to Patan Hospital Emergency Department in December 2018 with complaints of abdominal pain that had persisted for two days. She had multiple episodes of vomiting. In terms of the patient’s history, she had been treated with amlodipine and atenolol for hypertension and undergone a total abdominal hysterectomy 5 years prior for fibroids.”
  The detailed life style such as dietary habits and the nature of work that the patient was involved in, is not mentioned in the article. The article does not talk about the type of farming and her exposure to possible causative agents and is silent about the possible route of transmission of Cryptococcus to this patient.
  This would have been of great value because it is evident that Cryptococcus grows readily from soil contaminated with avian excreta, particularly that of pigeons as Cryptococcus can assimilate the xanthine, creatinine, urea and uric acid found in it. One of the possibilities of transmission may be this as the patient is a farmer and the Lalitpur area is home for many pigeons too. However, it is equally possible that the fungus might be transmitted via other sources such as vegetables, fruits and dairy products.
5. Regarding the issue of socio-political conditions and its interference in the management of this patient: The article says liposomal amphotericin B is expensive and unaffordable by the patient. But the article does not mention the price of that drug in Nepal. Discussing on the economic aspect without telling its price seems anecdotal than factual evidence.
  Similarly, what is the real socio-economic status of patient? Because the patient is a resident of the capital city and has undergone expensive investigations and treatment in Intensive Care Units of a tertiary level hospital. Also, having said the patient had political connection, how likely is the patient being poor? Which accepted socio-economic scale is being used for labeling the patient as poor.
  The article also says that, “The government of Nepal provides liposomal amphotericin B free of cost to patients with visceral leishmaniasis, and the government policy dictates use of the medicine in patients with visceral leishmaniasis only.” In this context it is important to note that visceral leishmaniasis is one of the Neglected Tropical Diseases (NTDs).

 It is important to discuss the following:

- Number of patient suffering from each of Visceral Leishmaniasis and Cryptococcal Meningitis.
- Socio-economic status of patients suffering those diseases.
- Cost-effectiveness of drug (liposomal amphotericin B) use relative to the differential dose required to treat Visceral Leishmaniasis and Cryptococcal Meningitis.
*For Visceral Leishmaniasis:*
*Single dose of 10 mg/kg (provided free of cost)*
*Total : 500 mg for 50 kg man/women*
*For Cryptococcal Meningitis:*
*In this case- 56 vials each of 50 mg*
*Total : 2800 mg*

Therefore it can be said that financial scheme or insurance policies may be a better way out. But in LMICs where Neglected Tropical Diseases are highly prevalent that should be more of hospital policies rather than Public Health policies at large.

 In conclusion, the case report has raised pertinent issues which could be an initiative of bridging the gap in hospital services guidelines and Healthcare Delivery System of countries. This report also very well highlighted that managerial issues could be barrier to life saving situations in an absence of clarity in service delivery policies.

 One more issue arises with this case report, that is the annual report of Department of Health Services Nepal needs to be equipped to do the periodic sentinel surveillance to monitor the further trend and record the cases of cryptococcal meningitis.This evidence could be utilized to convince the revision of drug availability policies.

We confirm that we have read this submission and believe that we have an appropriate level of expertise to confirm that it is of an acceptable scientific standard, however we have significant reservations, as outlined above.

# Response

**Published:** 2019-06-11

**Authors:** Sudeep Adhikari

**Affiliations:** Patan Academy of Health Sciences, Nepal

Thank you for the constructive feedbacks.

Below are the point by point responses to your comments. Relevant changes have been made in the first version of this article based on the responses below.

**Comment 1**

The title says: “Case Report: Cryptococcal meningitis in an immunocompetent patient in Nepal - challenges in diagnosis and treatment.” However, the discussion part mentions “Though subclinical immunodeficiency was not ruled out, we could say that she was apparently immunocompetent.”

In addition, the patient attended the hospital through emergency department and was diagnosed and treated initially for biliary colic and later developed signs of meningitis.

Thus, it will be appropriate to write ‘apparently immunocompetent’ in the title itself.

**Answer-**

The title has been changed- “Case Report: Cryptococcal meningitis in an apparently immunocompetent patient in Nepal - challenges in diagnosis and treatment”

**Comment 2**

Ethical Dilemma

The authors say that they felt helpless in the past when poor people with such fungal infections died without amphotericin B.(Discussion section-4 ^th^ paragraph). This raises the ethical dilemma as public funded hospital through government system has the provision to treat free of cost if treatable disease in Nepal. There is no explanation why there were no case report when the poor patients died due to non-availability of liposomal amphotericin. As this case report displays one successful management after many deaths, can it be said that if the patient did not have any political connection she would have died without liposomal amphotericin B and this case report would not have appeared?

**Answer-**

Yes our patient could potentially have died without political connection and access to liposomal amphotericin B and this has been included in the discussion section.

**Comment 3**

The article is focused on two issues:

a) Cryptococcal meningitis should be a diagnostic consideration even when dealing with meningitis in immunocompetent patients.

b) How sociopolitical conditions interfere with treatment in low- and middle-income countries (LMICs) and affect the health system of the country.

Though these two are presented as pertinent issues that affect the disease outcome.

**Comment 4**

The case report has missed few important points:

The case is described in the article as: “A 50 years old Aryan female farmer, from Lalitpur district, Nepal, presented to Patan Hospital Emergency Department in December 2018 with complaints of abdominal pain that had persisted for two days. She had multiple episodes of vomiting. In terms of the patient’s history, she had been treated with amlodipine and atenolol for hypertension and undergone a total abdominal hysterectomy 5 years prior for fibroids.”

The detailed life style such as dietary habits and the nature of work that the patient was involved in, is not mentioned in the article. The article does not talk about the type of farming and her exposure to possible causative agents and is silent about the possible route of transmission of Cryptococcus to this patient.

This would have been of great value because it is evident that Cryptococcus grows readily from soil contaminated with avian excreta particularly that of pigeons as Cryptococcus can assimilate the xanthine, creatinine, urea and uric acid found in it. One of the possibilities of transmission may be this as the patient is a farmer and the Lalitpur area is home for many pigeons too. However, it is equally possible that the fungus might be transmitted via other sources such as vegetables, fruits and dairy products.

**Answer**

In case presentation, changes have been made regarding her lifestyle - ‘She was a farmer living in suburban area of Lalitpur and involved in  growing of vegetables and rearing of cows and poultry.’

In discussion, this has been added - ‘Cryptococcus grows readily in soil contaminated with avian excreta particularly that of pigeons, and is transmitted to human via inhalation of the contaminated aerosol. One of the possibilities of transmission in our patient may be this as she is a farmer and the Lalitpur area is home for many pigeons too. However, it is equally possible that the fungus might have been transmitted via other sources such as vegetables, fruits and dairy products.

**Comment 5**

Regarding the issue of socio-political conditions and its interference in the management of this patient: The article says liposomal amphotericin B is expensive and unaffordable by the patient. But the article does not mention the price of that drug in Nepal. Discussing on the economic aspect without telling its price seems anecdotal than factual evidence.

Similarly, what is the real socio-economic status of patient? Because the patient is a resident of the capital city and has undergone expensive investigations and treatment in Intensive Care Units of a tertiary level hospital. Also, having said the patient had political connection, how likely is the patient being poor? Which accepted socio-economic scale is being used for labeling the patient as poor.

The article also says that, “The government of Nepal provides liposomal amphotericin B free of cost to patients with visceral leishmaniasis, and the government policy dictates use of the medicine in patients with visceral leishmaniasis only.” In this context it is important to note that visceral leishmaniasis is one of the Neglected Tropical Diseases (NTDs). 

It is important to discuss the following:

- Number of patient suffering from each of Visceral Leishmaniasis and Cryptococcal Meningitis.
- Socio-economic status of patients suffering those diseases.
- Cost-effectiveness of drug (liposomal amphotericin B) use relative to the differential dose required to treat Visceral Leishmaniasis and Cryptococcal Meningitis. *For Visceral Leishmaniasis:* *Single dose of 10 mg/kg (provided free of cost)* *Total : 500 mg for 50 kg man/women* *For Cryptococcal Meningitis:* *In this case- 56 vials each of 50 mg* *Total : 2800 mg*

Therefore it can be said that financial scheme or insurance policies may be a better way out. But in LMICs where Neglected Tropical Diseases are highly prevalent that should be more of hospital policies rather than Public Health policies at large.

In conclusion, the case report has raised pertinent issues which could be an initiative of bridging the gap in hospital services guidelines and Healthcare Delivery System of countries. This report also very well highlighted that managerial issues could be barrier to life saving situations in an absence of clarity in service delivery policies.

One more issue arises with this case report that is the annual report of Department of Health Services Nepal needs to be equipped to do the periodic sentinel surveillance to monitor the further trend and record the cases of cryptococcal meningitis. This evidence could be utilized to convince the revision of drug availability policies.

**Answer**

The details have been added in the discussion - ‘Nepal is an endemic region for VL with incidence of 0.11 cases per 10000 population. Each 50 mg vial of liposomal amphotericin B costs ranging 35- 50 dollars, but WHO has arranged each vial at subsidized rate of 20 dollars for the use for VL. The cost of treatment of a 50 kg patient would be 200 dollars for VL which has been provided free of cost with the help from WHO. Cryptococcal meningitis is not an uncommon infection in Nepal as compared to VL with estimated burden of 0.06 cases per 10000 population. But the expenditure for its treatment is almost 6 times more (1120 dollars for a 50 kg patient) as compared to VL even if we calculate using the WHO subsidized rate. So this has been a major hindrance in treatment of this fungal infection. We did not use any standard socioeconomic scale to determine if our patient was poor. However, based on the fact that the expenditure for complete treatment of cryptococcal meningitis exceeds the annual income of an average Nepalese person (Per Capita Income of Nepal is 800 dollars as of 2017), we assume that the inability of the family members to buy liposomal amphotericin B on their own is valid. Moreover the expensive investigations and stay in the Intensive Care Unit further hampered their ability to buy the required medicine as there is no point-of-care health insurance schemes for most patients including in our patient’s case.’
